# Different outcomes after proximal femoral replacement in oncologic and failed revision arthroplasty patients - a retrospective cohort study

**DOI:** 10.1186/s12891-021-04673-z

**Published:** 2021-09-22

**Authors:** Andreas Toepfer, Veit Straßer, Andreas Ladurner, Anna-Katharina Calek, Primoz Potocnik, Ruediger von Eisenhart-Rothe

**Affiliations:** 1 Department of Orthopaedic Surgery and Traumatology, Kantonsspital St. Gallen, Rorschacherstrasse 95, CH-9007 St. Gallen, Switzerland; 2 Department of Orthopaedics and Sport Orthopaedics, Technical University of Munich, 81547 Munich, Germany

**Keywords:** Proximal femoral replacement, Modular megaprosthesis, Failed revision arthroplasty, Malignant bone tumor, Musculoskeletal tumor, Limb salvage

## Abstract

**Background:**

Proximal femoral replacement (PFR) is a technically demanding procedure commonly performed to restore extensive, oncological or non-oncological bone defects in a severely debilitated patient collective. Depending on different indications, a varying outcome has been reported. The aim of the study was to assess the functional outcomes and complication rates of PFR with the modular Munich-Luebeck (MML) femoral megaprosthesis (ESKA/Orthodynamics, Luebeck, Germany), and to highlight outcome differences in patients treated for failed revision total hip arthroplasty (THA) or malignant bone disease.

**Methods:**

A retrospective review of patients treated with PFR for failed THA or malignant tumor disease between 2000 and 2012 was performed. Patient satisfaction, functional outcome (VAS, SF-12, MSTS, WOMAC, TESS), complications and failure types (Henderson’s failure classification) were assessed. A Kaplan-Meier analysis determined implant survival.

**Results:**

Fifty-eight patients (age: 69.9 years, BMI: 26.7 kg/m^2^, mean follow-up: 66 months) were included. The mean SF-12 (physical / mental) was 37.9 / 48.4. MSTS averaged 68% at final follow-up, while mean WOMAC and TESS scored 37.8 and 59.5. TESS and WOMAC scores demonstrated significantly worse outcomes in the revision group (RG) compared to the tumor group (TG). Overall complication rate was 43.1%, and dislocation was the most common complication (27.6%). Implant survival rates were 83% (RG) and 85% (TG; *p* = n.s.) at 5 years, while 10-year survival was 57% (RG) and 85% (TG, *p* < 0.05).

**Conclusions:**

PFR is a salvage procedure for restoration of mechanical integrity and limb preservation after extensive bone loss. Complications rates are considerably high.

Functional outcomes and 10-year implant survival rate were worse in the RG compared to the TG. Strict indications and disease-specific patient education are essential in preoperative planning and prognosis.

## Background

Advances in imaging, surgical techniques and (neo-)adjuvant therapy have led to improved survival rates in malignant tumor disease in recent decades [1]. Megaprostheses have become an integral part in treating extensive bone defects, including malignant bone disease, in the vicinity of large joints [2–4]. Alongside other surgical options like biological auto- and allograft reconstruction or custom made prostheses, modular megaprostheses can restore the mechanical integrity of the affected joint and limb, facilitate weight bearing and preserve the patient’s ability to walk [5–7]. Compared to allograft prosthetic composites, proximal femoral replacements (PFR) are deemed to be less technically demanding and do not require biologic healing of the allograft-host interface [8, 9]. In addition, modular megaprostheses offer intraoperative flexibility and procedural standardization, and are advantageous due to their constant availability [6]. With the use of PFR, hip disarticulations and amputations for malignant bone tumors can be prevented in many cases, and limb-salvage procedures have become increasingly successful in recent decades [1, 5].

Similar to treating malignant bone tumors by wide resection, repeated failed total hip revision arthroplasty can lead to extensive bone defects, which often can not be restored with standard revision implants. In these cases, the use of PFR may offer an opportunity in restoring otherwise non-reconstructable, non-oncological bone defects [7, 10]. This aspect may become increasingly important in the near future, as demographic changes lead to continuously increasing numbers of primary and also revision total hip arthroplasties [11].

The clinical, radiographic and patient-reported outcome of megaprostheses have been discussed in the literature over the last decades [5, 9, 10, 12, 13], but a discrimination of outcomes according to the varying indication for PFR hasn’t been addressed in the past. In recent years, literature focusing on PFR results in revision total hip arthroplasty for severe femoral bone loss has emerged [2–4, 14].

The purpose of this study was to assess the functional outcomes and complication rates of PFR performed with the Modular Munich-Luebeck (MML) femoral megaprosthesis (ESKA/Orthodynamics, Luebeck, Germany), and to compare the results for this procedure in patients treated for failed revision hip arthroplasty or malignant bone disease.

## Methods

The investigation has been performed at a single orthopedic university tumor center. Institutional review board approval and written consent from each patient were obtained. The institution’s database was retrospectively reviewed. All patients undergoing PFR with the modular MML prosthesis (ESKA/Orthodynamics, Luebeck, Germany) for failed revision total hip arthroplasties or aggressive tumorous lesions between January 2000 and December 2012 were considered for study inclusion. Patients unwilling or unable (e.g. due to dementia) to participate and those deceased or not contactable at the time of investigation were excluded from further analysis.

A review of electronic and non-electronic patient’s charts and functional investigation of all eligible patients was performed. In addition, all patients were contacted by mail for outcome assessment, and a phone interview was conducted for assessing complications. Included patients were subdivided into a revision group (RG) or a tumor group (TG) according to the indication for PFR.

Demographic data of interest included age, gender, BMI, comorbidities, and indication for surgery.

### Implant specifications and surgical technique

**Fig. 1:**
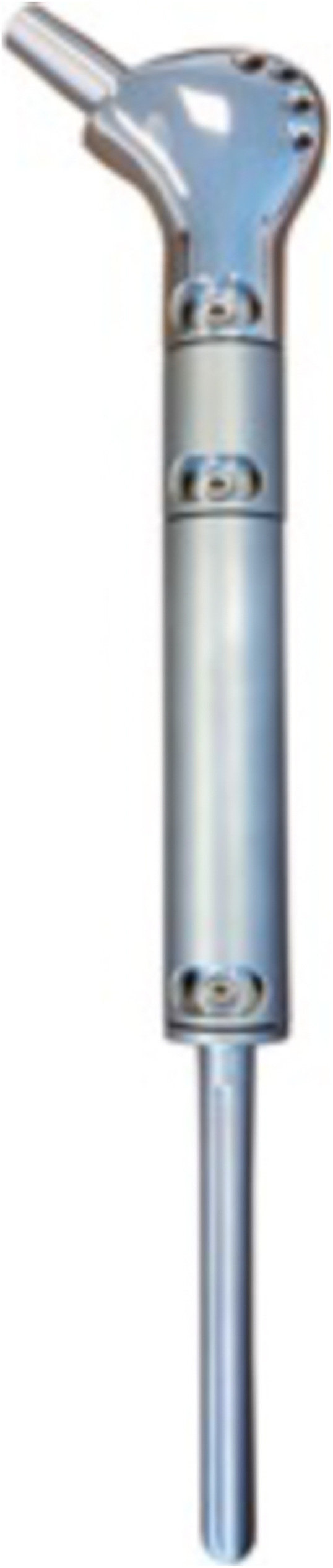
Modular Munich-Luebeck (MML) femoral megaprosthesis

**Fig. 2:**
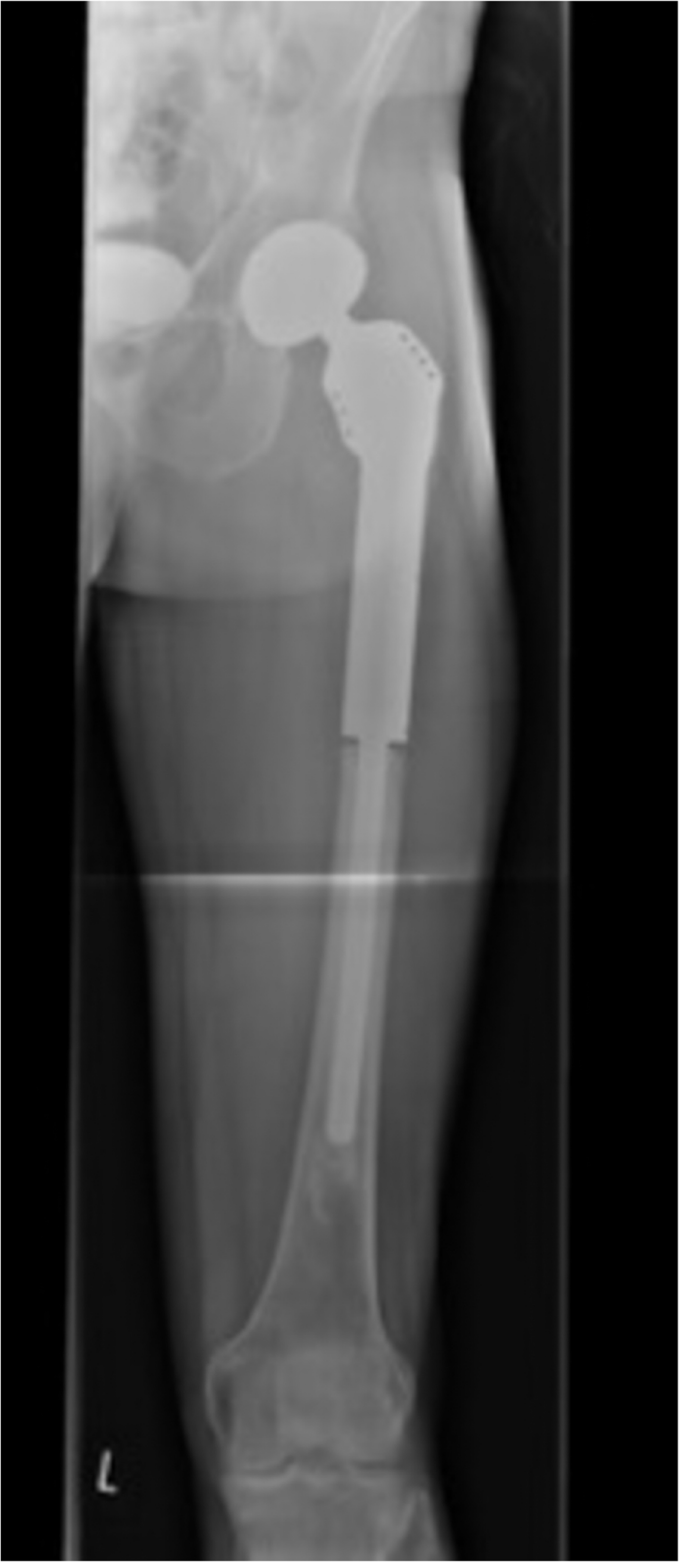
X-ray showing the Modular Munich-Luebeck (MML) femoral megaprosthesis in situ

The Modular Munich-Luebeck (MML) femoral megaprosthesis (ESKA/Orthodynamics, Luebeck, Germany) system comprises a proximal trochanteric module with a 12/14 mm taper, that allows for fixation of the abductor apparatus, extension modules and a curved or straight diaphyseal stem (Figs. 1 and 2). The extension modules are available in different lengths of 10 mm increments, with tapered connections and additional screw-locks, allowing rotational adjustments of ±15°. The 160 mm diaphyseal stem comes in different diameters of 11 to 16 mm. A cemented or cementless stem option is available, and both were used in this series. PFR was routinely coupled to a cementless primary cup as a standard, and a metal or ceramic head (32 mm or 36 mm) was used depending on the surgeon’s preference.

Dual-mobility cups (Versafit, Medacta, Castel San Pietro, Switzerland) were inserted according to the surgeons’ decision in selected cases after 2010. PFR hemiarthroplasty with a bipolar head was performed in the (rare) occasion of very old and frail patients, in order to reduce surgical trauma and surgery time. The hip abductors were reinserted to the trochanteric module using non-absorbable suture material, and a hip orthosis (Newport hip orthosis, Orthomerica, Orlando (FL), USA or Coxa Top orthosis, Adev, Luebeck, Germany) was applied to all patients for the first 6 to 12 weeks following surgery in an attempt to reduce (early) dislocation rates.

### Outcome parameter

Patient satisfaction was evaluated on a 6-point Likert-scale (1 = very good, 6 = very bad), and all patients were asked whether they would recommend the procedure to friends or relatives. Clinical outcome was evaluated using a visual analogue scale (VAS), the short form-12 Health-Survey (SF-12), the Musculoskeletal Tumor Society Score (MSTS), the Western Ontario and McMaster University Osteoarthritis Index (WOMAC) and the Toronto Extremity Salvage Score (TESS).

The VAS [15] is a psychometric, unidimensional measure of pain intensity, which has been widely used in diverse adult populations. It is the tool most commonly used to quantify pain at the reporting institution.

The SF-12 [16] is a self-reported outcome measure assessing the impact of health on an individual’s everyday life. It is designed as a general measure of health and often used as a quality of life measure.

The MSTS [17] is an established tool to assess functional outcome and quality of life following limb preserving tumor resection, and has recently been applied for non-tumor associated megaprosthesis implantation such as in failed revision hip arthroplasty [7, 10]. It consists of six domains, scored on a 0 to 5 scale and transformed into an overall score ranging from 0 to 100% with a higher score indicating better function.

The WOMAC [18] is a widely used three-dimensional patient-reported outcome instrument consisting of 24 questions that are linearly transformed to a 0–100 scale with higher scores indicating more severe impairment. The score has been extensively tested for validity, reliability, feasibility and responsiveness [18, 19].

The TESS [20] is a self-administered questionnaire that includes 30 items regarding activity limitations in daily life, such as restrictions in body movement, mobility, self-care, and performance of daily tasks and routine. It is widely used for the functional assessment of patients following limb salvage surgery. The degree of physical disability is rated from 0 (not possible) to 5 (without any problem). The raw score is converted to a score ranging from 0 to 100 points, with higher scores indicating less functional limitations.

Complications were recorded according to Henderson’s failure mode classification for tumor endoprostheses [12], that categorizes failure modes into mechanical or non-mechanical failures, and distinguishes five different types of failure (Table 1). As demonstrated recently by other authors [4, 10, 21], the Henderson classification, originally intended for a failure mode assessment of megaprostheses after tumor resection, is deemed suitable for non-oncological indications as well.

**Table 1:** Henderson’s classification [12]

General category	Classification	Mode of failure	Description
Mechanical	Type I	Soft-tissue failure	Limited function owing to insufficient musculo-ligamentous attachment (e.g., instability, tendon rupture, aseptic / superficial wound dehiscence
	Type II	Aseptic loosening	Clinical and radiological evidence of peri-prosthetic loosening
	Type III	Structural failure	Breakage, fracture or wear related failure resulting in deficient supporting structure
Non-mechanical	Type IV	Infection	Infected reconstruction not amenable to retention
	Type V	Tumor progression	Recurrence or progression of tumor with endoprosthesis contamination

Implant survival was assessed using a Kaplan-Meier curve, and two types of mega-prosthesis failure were defined. Type A failure occurred when complete implant removal had to be performed, i.e. when the diaphyseal stem had to be revised (e.g. stem breakages) or hip disarticulation to be performed. Type B failure was determined by revision with (partial) implant retention. This included revision for hematoma, dislocation, periprosthetic infection (without the need for diaphyseal stem removal), or structural failure of the prosthesis (excluding stem breakages).

### Statistics

The mean value, standard deviation and minimum/maximum values were indicated where applicable. Survivorship analysis was performed using the Kaplan-Meier survivorship method. Chi-squared test was used to compare two groups with two categories. Where applicable, the Mann-Whitney *U* test for independent samples was used. Correlations between numerical data were performed with linear regression analysis, and Pearson’s correlation coefficient (*r*) is reported. *p* values were two-sided and considered statistically significant if smaller than 0.05. Statistical analysis was performed using SPSS 2.0 (IBM, Armonk, NY, USA).

## Results

### Patient demographics

A total of 165 eligible patients were identified (female/male: 101/64). 97 of the 165 patients were deceased at the time of follow-up. Ten patients had to be excluded from our study, as 5 patients had moved away and 4 patients declined to participate. One patient was unable to attend due to advanced dementia. Thus, a total of 58 patients (58 implants) were included in our study. Thirty-one patients (mean age 76,2 ± 9,4 years (range 49–92)) were assigned to the RG, while the TG consisted of 27 patients (mean age 62,9 ± 16,7 years (range 22–93)). 83.9% in the RG and 55.6% in the TG were female (overall: 70.1% female). The overall mean BMI was 26.7 kg/m^2^ and did not significantly differ in between the treatment groups. The right side was affected in 30/58 cases (RG: 18, TG: 12). The mean follow-up time was 52.2 ± 36.6 months (range 11–160 months). Relevant comorbidities where found in 62.1% of patients overall. Here, the RG showed a significantly higher prevalence (RG: 80.6%, TG: 40.7%, *p* < 0.001). The most common comorbidity was hypertension, followed by cardiac arrhythmia and diabetes. Patient demographics and a thorough listing of the comorbidities are given in Table 2.

**Table 2:** Patient demographics and comorbidities

		Overall	RG	TG	***P***
**Patients**	N	58	31	27	
**Sex**	f / m	41 / 17	26 / 5	15 / 12	
**Age (years)**	Mean (min – max)	69,9 ± 14,9 (22–93)	76,2 ± 9,4* (49–93)	62,9 ± 16,7* (22–93)	*p < 0.05
**BMI (kg/m**^**2**^**)**	Mean ± SD (min – max)	26,7 ± 4,3 (20.9–39.1)	27,3 ± 4,3 (21,3 - 39,1)	24,9 ± 3,9 (20,9 - 36,5)	*p* = n.s. (0.097)
**Affected side**	right / left	30 / 28	18 / 13	12 / 15	
**Follow-up (months)**	Mean (min – max)	52.2 (11–160)	49,0 (11–139)	55,9 (14–160)	
**Comorbidities**					
- Patients	N (%)	36 (62.1%)	25 (80.6%)*	11 (40.7%)*	*p < 0.001
- Hypertension		22	15	7	
- Diabetes		7	4	3	
- COPD		3	2	1	
- Congestive heart failure		4	3	1	
- CAD		2	2	–	
- Cardiac arrhythmia		8	4	4	
- Hypothyroidism		5	3	2	
- Chronic renal failure		3	3	–	
- Neoplasia		4	2	2	
- Decubitus		1	1	–	
- Arthrosis		2	2	–	
- Dementia		2	2	–	
- Depression		1	1	–	
- Schizophrenia		1	1	–	
- Cerebrovascular insult		1	1	–	
- Epilepsia		1	–	1	

*RG* Revision group, *TG* Tumor group, *f* Female, *m* Male, *BMI* Body-mass-index, *COPD* Chronic obstructive pulmonary disease, *CAD* Coronary artery disease

Indication for PFR in the RG were periprosthetic joint infection (PJI) in 12 and periprosthetic fractures in 11 cases, while aseptic loosening and mechanical failure of a prior THA associated with relevant osseous defects were reasons to revise 6 and 2 patients to a PFR, respectively. Overall, 43.4% of patients had their THA revised more than once before undergoing PFR. The implant removed during surgery was a revision THA stem in 18 cases, a THA in conjunction with LISS plate (DePuy-Synthes, Oberdorf, Switzerland) in 2, and a PFR in one case. On 10 occasions, PFR was performed in patients with prior Girdlestone resection arthroplasty for PJI.

In the TG, 10 primary (bone sarcomas) and 14 secondary malignancies (metastases) of the bone, two soft tissue sarcomas with involvement of the adjacent bone (1 angiosarcoma, 1 fibromyxosarcoma) and 1 recurrence of a giant cell tumor were responsible for extensive bone destruction of the proximal femur not reconstructable with standard implants. The 10 primary bone malignancies were composed of 7 chondrosarcomas, 2 osteosarcomas and 1 Ewing sarcoma. Bony metastases originated from breast cancer in 6 cases, from a renal cell carcinoma or prostate cancer in 3 cases each, or from a thyroid and bronchial carcinoma (two cases each).

### Patient satisfaction

Overall, patient satisfaction averaged 2.7 (RG: 3.0, TG:2.5) on a 6-point Likert-scale (1 = very good, 6 = very bad) and did not differ in between the treatment groups. 42.8% in the RG and 53.8% in the TG rated their satisfaction “very good” (i.e. 6/6 points) or “good” (i.e. 5/6 points). None of the patients in either group reported a “very bad” result (i.e. 1/6 points). 76% of all patients would recommend the treatment to relatives or friends, but 33% of patients in the RG and 9% of patients in the TG would not (see Table 3 for details).

**Table 3:** Patient satisfaction and clinical outcome

		Overall	RG	TG	***P***
**Patient satisfaction**					
- (6-point Likert scale)	Mean (min. max)	2.7 (1–5)	3.0 (1–5)	2.5 (1–5)	*p* = n.s.
- Recommendation° “no”	%	19.6%	33%*	9%*	*p < 0.05
**VAS**	Mean				
- preoperative		5	5	6	*p* = n.s.
- postoperative		3	3	3	*p* = n.s.
**SF-12**	Mean ± SD				
- physical subdomain		37.9 ± 9.2	37,7 ± 9,6	38,1 ± 8,8	*p* = n.s.
- mental subdomain		48.4 ± xx	47,2 ± 10,4	49,8 ± 12,1	*p* = n.s.
**MSTS numerical**	Mean ± SD (min – max)	17,0 ± 6,0 (3–28)	15,3 ± 5,6 (3–27)	18,7 ± 5,0 (11–28)	*p* = n.s. (0.056)
**MSTS %**	Mean	68.0%	61.2%	74.8%	
**WOMAC total**	Mean ± SD	37,8 ± 19,5	47,2 ± 21,1*	30,0 ± 14,1*	*p < 0.05
- WOMAC pain		4,6 ± 4,5	6,2 ± 5,5	3,3 ± 2,9	*p* = n.s.
- WOMAC stiffness		2,1 ± 2,0	2,7 ± 2,3	1,7 ± 1,7	*p < 0.05
- WOMAC ADL		31,1 ± 15,1	38,4 ± 15,7*	25,0 ± 11,8*	
**TESS**	Mean ± SD (min – max)	59.5 ± 22.2 (16–115)	49.5 ± 21.9* (16–115)	67.6 ± 18.2* (37–110)	*p < 0.05

*RG* Revision group, *TG* Tumor group, *VAS* Visual analogue scale, *SF-12* Short form-12 Health-Survey, *MSTS* Musculoskeletal Tumor Society Score, *WOMAC* Western Ontario and McMaster University Osteoarthritis Index, *TESS* Toronto Extremity Salvage Score, *SD* Standard deviation

° ”would you recommend the treatment to relatives or friends”

### Clinical outcome

A thorough listing of the clinical outcome is given in Table 3. The overall mean VAS improved by 2 points from 5 (preoperatively) to 3 following PFR. 22.6% (RG) and 11.1% (TG) of patients reported deteriorated pain-levels following the procedure (Table 3).

Quality of life as assessed by the SF-12 averaged 37.9/48.4 (physical/mental subdomain). No significant difference in between the treatment groups were found in both the physical (37.7 (RG) vs 38.1 (TG)) and mental subdomains (47.2 (RG) vs. 49.8 (TG)).

The mean MSTS score across both groups was 17,0 ± 6,0 (range 3–28; MSTS% 56,2%). The numbers in the RG (15,3 ± 5,6 (3–27; 51.0%)) did not differ significantly from values in the TG (18,7 ± 5,0 (11–28; 62,3%)), but a tendency in favor of the TG was found. Sub-domain analyses revealed a significant difference between the treatment groups in the need for walking supports, with patients in the RG having to use crutches significantly more frequently than patients in the TG.

Regarding WOMAC, the RG scored higher than the TG in all categories, indicating worse postoperative quality of life for the RG. Overall, WOMAC total averaged 37.8 points (RG: 47.2 ± 21,1, TG: 30.0 ± 14.1, *p* < 0.05).

The mean postoperative TESS was 59.5 points. The RG scored significantly worse in the overall TESS (49.5 (RG) vs. 67.6 (TG), *p* < 0.05). Most patients in both groups had difficulties with kneeling, getting up from a kneeling position, or performing sporting activities (Table 3).

### Complications

25/58 (RG: 15, TG: 10) patients (43.1%) suffered from a total of 38 complications within the given follow-up period. Overall, there were 30 implant-related complications (type I to IV failures according to Henderson’s classification), while no tumor recurrence / progression (type 5 failure) was seen. A thorough listing of the implant related complications is provided in Table 4.

**Table 4:** *Complications* according to Henderson’s failure mode classification [12]

General category	Classification	Mode of failure	Overall N(p)/N(c) (%(p))	RG N(p)/N(c) (%(p))	TG N(p)/N(c) (%(p))
Mechanical	Type I	Soft-tissue failure	16/21 (27.6%)	10/15 (32.3%)	6/6 (22.2%)
	Type II	Aseptic loosening	2/2 (3.4%)	1/1 (3.2%)	1/1 (3.7%)
	Type III	Structural failure	3/3 (5.8%)	1/1 (3.2%)	2/2 (7.4%)
Non-mechanical	Type IV	Infection	4/4 (7.7%)	3/3 (9.7%)	1/1 (3.7%)
	Type V	Tumor progression	–	–	–
**TOTAL**	**ALL TYPES**		25/30 (43.1%)	15/20 (48.4%)	10/10 (37.0%)

*N(p)* Number of patients, *N(c)* Number of complications

Type I failures (soft tissue failures) were observed only in the form of (recurrent) hip dislocations in 16 patients (RG:10, TG:6). Only conventional cups with either a 32 mm or 36 mm head were affected by dislocation, while this was not observed with dual mobility cups.

Type II failures (aseptic loosening) occurred in one case of each group. In both cases, loosening concerned a cemented diaphyseal stem.

One patient of the RG and two patients in the TG sustained structural failure (stem breakage, type III failure) of their PFR 14, 28 and 32 months postoperatively. A single ceramic head breakage was not classified within the Henderson classification analysis as structural failure of the PFR, since the head is not an inherent part of the modular megaprosthesis itself.

Four cases (RG:3, TG:1) of periprosthetic joint infection (Type IV failure) were observed and underwent revision. In the RG, two infections occurred in patients treated for aseptic loosening of the prior implant 12 and 28 months after PFR. The third infection was diagnosed in a patient with prior Girdlestone resection arthroplasty for periprosthetic joint infection 8 months after PFR. According to the pathogen and the course of time, a hematogenous bacterial spread rather than a persistent infection had to be assumed.

Eight additional complications (RG:7, TG:1) were classified as “unspecific” and therefore not attributable to any of Henderson’s failure mode classification:

In the RG, these were surgical side hematoma in three cases, seroma in two, and pulmonary embolism and ceramic head breakage in one case each. 5/7 complications required a single revision each. The only unspecific complication observed in the TG was one compartment syndrome following the surgical intervention that underwent revision.

Overall, the reported complications resulted in 25 revision surgeries in 19 patients (RG:12, TG:7), which are outlined in detail in Table 5.

**Table 5:** Reason for revision and implant failure with respect to type A or type B failure. Individual cases shown. N(p), number of patients; N(r), number of revisions

	RG	TG
**N (p) / N (r)**	12 / 18	7 / 7
**Reason for revision / individual cases**	• Rep. dislocation^b^ • Rep. dislocation^b^ • Loosening^a^ • Implant breakage^a^ • Hematoma^b^ • Hematoma^b^ • Infection^a^ • Rep. dislocation / hematoma^b^ • Rep. dislocation / seroma^b^ • Rep. dislocation / infection^b^ • Rep. dislocation / rep. Dislocation^b^ • Ceramic head breakage^b^ / rep. Dislocation^b^ / Infection^a^	• Rep. dislocation^b^ • Rep. dislocation^b^ • Loosening^a^ • Implant breakage^a^ • Implant breakage^a^ • Compartment syndrome^b^ • Infection^b^

^a^ type A failure

^b^type B failure

### Implant survival

**Fig. 3:**
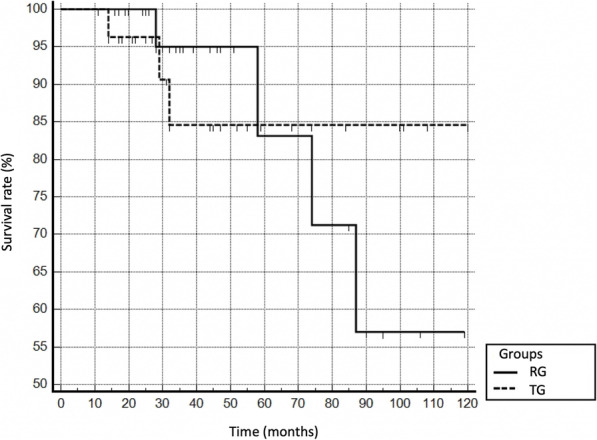
Kaplan-Meier survival analysis: PFR survival with respect to type A failure (complete implant removal). RG, revision group (continued line); TG, tumor group (dotted line)

Overall, 5-year implant survival (type A failure) was 83% in the RG and 85% in the TG, while a 10-year implant survival rate of 57 and 85% was observed, respectively. Seven cases were revised across the treatment groups (see Table 5). In the RG, this endpoint was observed in 4/31 cases (12.9%) at an average of 66 (28–87) months post-surgery. The reason for type A failure was periprosthetic infection in two cases, while aseptic loosening and structural implant failure (stem breakage) warranted implant removal in one case each. In the TG, type A implant failure was detected in 3/27 cases (11.1%) after a mean follow-up of 29 [14–32] months. Two diaphyseal stem breakages and one aseptic loosening were observed. The Kaplan survival analysis for type A failures is given in Fig. 3.

**Fig. 4:**
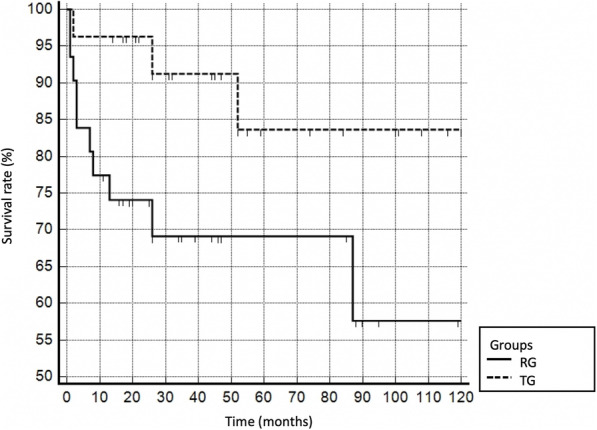
Kaplan-Meier survival analysis. PFR implant survival with respect to dislocations, periprosthetic infections (stem preserved), hematomas, and breakage of the prosthesis (diaphyseal stem breakages excluded). RG, revision group (continued line); TG, tumor group (dotted line)

Type B failures occurred in 14 patients (RG = 10, TG = 4) at a mean of 5 months (range 0–87 months) after surgery. In 7 cases (5 revision patients, 2 tumor patients) soft tissue failure such as recurrent dislocations and wound healing problems led to a type B failure, while hematoma and structural failure were the reason for failure in 3 and 1 RG patients, respectively. In both groups (RG and TG), one failure due to deep infection (treated without removal of the stem) was detected. Figure 4 shows the survival curve of the MML prosthesis for type B failures in RG an TG patients.

A group comparison showed higher revision rates for patients of the RG, and the PFR had to be exchanged in 32.3% after a mean of 5 months (range 1–87 months). In the TG, 14.8% revisions after an average follow-up of 14 months (range 0–52 months) were observed.

## Discussion

PFR is an established treatment option to restore femoral integrity and provide limb salvage [4, 6, 22–24] in massive bone defects of the proximal femur due to oncologic conditions or failed repeated revision arthroplasty. In the presented study, clinical and functional outcome following 58 PFRs using the modular MML prosthesis (ESKA/Orthodynamics, Luebeck, Germany) were reported, and outcome differences in patients treated for malignant bone disease or failed revision total hip arthroplasty were highlighted.

The main finding of the study is that the overall functional outcome was significantly worse in the RG compared to the TG, including the need for walking supports (RG: 86%, TG: 48%). Interestingly, Toepfer et al. [25] reported the same findings in patients undergoing total femoral replacement for non-oncologic or oncologic indications. The overall mean WOMAC score in this series was 37.8, which is consistent with values reported by Al-Taki et al. [6], who also found postoperative SF-12 scores very similar to ours (physical subdomain: 37.0 vs. 37.9 (present study), mental subdomain: 50.8 vs 48.4 (present study)). Functional outcome measurement with the MSTS score revealed satisfying (RG) to good (TG) mean scores in our series. Compared to the current literature presenting results with a consistent range between 60 and 88% for neoplastic and non-neoplastic indications [7, 26], our own results are very similar (overall score: 68.0%, RG: 61,2%, TG: 74.8%).

Regarding the TESS score, both treatment groups showed bad results due to limitations in sports activity and impaired leisure activity. Further analysis of the TESS and WOMAC score demonstrated an impaired function within daily activities in non-oncologic patients with a significantly poorer outcome (TESS; WOMAC). Our results showed no relevant difference in pain reduction pre- and postoperative between the two groups. Although, tumor patients were more likely satisfied (*p* = n.s.) and would rather recommend the procedure (*p* < 0.05).

The inferior functional outcome in the RG is potentially related to different patient demographics and the ever deteriorating health condition following multiple revision surgeries. In the RG, patients were significantly older (76.2 vs. 62.9 years, *p* < 0.05), and suffered significantly more often from additional medical conditions (e.g. renal failure, diabetes, coronary heart disease). The number of previous surgical procedures was inherently higher, and a compromised function of the hip abductors and other muscles resulting from prior surgical procedures cannot be restored by another revision.

Another main finding of the present study was the high overall complication rate in patients treated with PFR of 43,1%. In 32.7%, revision was necessary due to implant failures. An overall complication rate of 32.2% was reported by Vaishya et al. [4] in a review of PFR in a non-neoplastic patient population, while De Martino et al. [2] reported a reoperation rate of 22% after a mean follow-up of 5 years.

Major complications of PFR reported in the literature are instability, infection and aseptic loosening, with dislocation being the most common reported complication after femoral megaprosthesis reconstruction regardless of the indication [2, 4, 12, 14, 22, 27, 28]. Matching the findings of the literature, hip dislocation was found the most common complication in our series as well. This also represented the most common cause for revision irrespective of the treatment group. Overall, the dislocation rate was 27.6%, and dislocations made up 64% of all implant-specific complications. Dislocation rates reported in literature vary between 4 and 42% [2, 22, 24, 29]. Papers reporting low dislocation rates [2, 29] commonly use constrained liner or dual mobility cups. Due to their promising results [1, 10], dual mobility cups were used on 7 occasions in our patient collective after 2010. None of these patients sustained a dislocation 14 to 21 months postoperatively.

Risk factors for instability include age, multiple previous surgeries often resulting in a compromised soft tissue envelope, inability to achieve secure repair of the abductors or secure fixation of the prosthesis to the remaining host bone, and inappropriate soft tissue tension [9, 12, 14, 28]. Hence, Parvizi et al. [28] high-lighted the role of different factors in the reduction of the risk of dislocation in this type of reconstruction, including restoration of length using modular components, reattachment of soft tissue and /or remaining trochanteric bone around a porous proximal component, and the use of constrained liners. However, longer-term follow-up is needed to assess the success of constrained liners in the setting of PFR [28]. The postoperative use of hip orthosis is discussed controversially [9, 24].

Periprosthetic joint infection and aseptic loosening were found in 16% (4 cases) and 8% (two cases of a cemented stem) in our series, respectively. The proportion of patients with infectious complications in our study was comparable to the number reported by Grammatopoulos et al. [29], while similar numbers for aseptic loosing were described by Viste et al. [3].

Biomechanically, the long lever arm of the lower limb exposes the diaphyseal stem to high torsional and compressive stresses, predisposing early loosening. Due to heterogenic patient populations, different implant types and retrospective study designs, it is still unclear if cemented or press-fit diaphyseal stems show better results regarding implant survival in megaprostheses [1, 3, 7, 21, 30]. Investigations performed by Pala et al. [31], Gebert et al. [32] and Oliva et al. [33] suggested favourable results in cementless prostheses, whereas Gerdemeyer et al. [30] and Toepfer et al. [21] found fewer cases of aspetic loosening in cemented megaprostheses of the distal femur in their case series.

Tumor recurrence was reported to occur in 4% after a mean evaluation period of 47 months in the study by Henderson et al. [12]. No recurrence was found in our series at a follow-up period of 52,2 ± 36,6 months, emphasizing the importance of adequate resection margins.

In our series, severe complications leading to implant removal within the first 5 years were found in both groups to the same extent (5-year survival rate: 83% RG, 85% TG), while the 10-year implant survival rate was significantly higher in the TG (RG: 57%, TG: 85%). The literature reports 5-year survival rates of 31–96% [4, 9, 22, 34]. Vaishya et al. [4] reported a mean implant survival rate of 80% after 5 years in his review article on PFR for failed revision arthroplasty. Thus, the survival rates found in this series are comparable to the data reported in previous studies.

This study has several limitations. First, the retrospective study is based on patients with diverse demographic characteristics treated by various surgeons. As a result, the study suffers from methodological difficulties inherent to retrospective investigations and is subject to recall- and selection bias. Second, the final sample size is small because the nature of this procedures is infrequent and performance of statistical analysis is thereby difficult. Ninety-seven patients had deceased by the time of the investigation, and further 10 patients were lost to follow-up. Our results potentially represent a best-case scenario, as patients lost to follow-up might be performing worse. Third, the given follow-up period might not be appropriate to make proper assumptions regarding the long-term outcome of megaprostheses, because many complications can occur after years [3, 4]. The results cannot be directly compared with other types of megaprostheses or biologic reconstructions and the treatment effect cannot be assessed due to a lack of preoperative functional scores.

## Conclusions

PFR enables limb salvage with the preservation of the structural integrity of the femur and allows for immediate stability for ambulation.

The study supports the use of PFR as an end-stage therapeutic option for extensive proximal femoral bone loss. Overall functional outcome measurements revealed satisfying results in both the oncologic and non-oncologic treatment group. However, functional outcomes as reported by TESS and WOMAC scores were significantly worse in the RG compared to the TG. This information is clinically important when counseling patients undergoing PFR for failed revision THA, as establishment of realistic expectations towards the outcome of the treatment can be regarded as an important aspect of modern healthcare. The incidence of implant-related complications and failures in PFR is considerably high, pointing out the risks of this procedure. Complication rates of 48% (RG) and 37% (TG) were observed, and dislocations were a major concern in this series. As candidates for this procedure are usually severely debilitated and their associated perioperative morbidity is high, strict indications are essential and potential burdens of the procedure have to be evaluated individually. The survivorship of the implant is acceptable, and 5-year implant survival rates of 83% (RG) and 85% (TG) were observed. However, only 57% of RG patients had an uneventful implant survival 10 years after surgery.

## Data Availability

The datasets used and/or analyzed during the current study are available from the corresponding author on reasonable request.

## Abbreviations

ADL: Activities of Daily Life
BMI: Body Mass Index
EbM: Evidence based Medicine
MML: “Modular (System) Munich-Lubeck” megaprosthesis
MSTS: Musculoskeletal Tumor Society Scoring System
PFR: Proximal femoral replacement
PJI: Periprosthetic joint infection
QOL: Quality of Life
RG: Revision group
TFR: Total femoral replacement
THA: Total hip arthroplasty
TG: Tumor group
SF-12: Short Form-12 Health Survey
TESS: Toronto Extremity Salvage Score
WOMAC: Western Ontario and McMaster Universities Arthritis Index
